# Targeting Immunometabolism in Glioblastoma

**DOI:** 10.3389/fonc.2021.696402

**Published:** 2021-06-16

**Authors:** Aditya A. Mohan, William H. Tomaszewski, Aden P. Haskell-Mendoza, Kelly M. Hotchkiss, Kirit Singh, Jessica L. Reedy, Peter E. Fecci, John H. Sampson, Mustafa Khasraw

**Affiliations:** Preston Robert Tisch Brain Tumor Center at Duke, Department of Neurosurgery, Duke University Medical Center, Durham, NC, United States

**Keywords:** glioblastoma, immunotherapy, metabolism, immunometabolism, tryptophan, arginine, 2HG, adenosine

## Abstract

We have only recently begun to understand how cancer metabolism affects antitumor responses and immunotherapy outcomes. Certain immunometabolic targets have been actively pursued in other tumor types, however, glioblastoma research has been slow to exploit the therapeutic vulnerabilities of immunometabolism. In this review, we highlight the pathways that are most relevant to glioblastoma and focus on how these immunometabolic pathways influence tumor growth and immune suppression. We discuss hypoxia, glycolysis, tryptophan metabolism, arginine metabolism, 2-Hydroxyglutarate (2HG) metabolism, adenosine metabolism, and altered phospholipid metabolism, in order to provide an analysis and overview of the field of glioblastoma immunometabolism.

## Introduction

Advances in immunotherapies have revolutionized cancer care, yet unfortunately, they have been largely unsuccessful in managing glioblastoma. One of the primary obstacles in treating glioblastoma with immunotherapy has been overcoming the heterogeneous and immunosuppressive tumor microenvironment (TME) that is, at least in part, regulated by tumor metabolism. Since 1927, when Otto Warburg et al. first described tumor’s preferential use of glycolysis to generate adenosine triphosphate (ATP) (1), there has been a burgeoning interest in understanding tumor metabolism and how it influences tumor growth. However, it is only recently that our understanding of tumor metabolism has extended beyond the confines of the tumor cell membrane and that we have begun to understand how tumor metabolism affects noncancerous cells such as tumor-infiltrating immune cells.

While glioblastoma cells are metabolically distinct from noncancerous tissue in the brain, certain metabolic similarities exist between glioblastoma and proliferating immune cells. These similarities include an upregulation of glucose utilization, glycolysis, fatty acid oxidization, amino acid metabolism, and nucleotide synthesis. As such, glioblastoma cells can induce immunosuppression by outcompeting immune cells for critical nutrients. In addition to contending with immune cells for metabolites, certain glioblastoma cells can also avail distinctive metabolic pathways to produce unique metabolites such as 2-Hydroxyglutarate (2HG) and extracellular adenosine, which can directly suppress the immune system. While there are multiple mechanisms by which tumors can alter their metabolism and influence the immune system, we have utilized large-scale omics analysis to selectively highlight pathways that are critical to glioblastoma pathogenesis (2–5).

In this review, we outline how altered metabolic pathways in glioblastoma contribute to immunosuppression and discuss approaches to target these phenomena, in order to improve future immunotherapy outcomes.

## Hypoxia

Hypoxia is a key feature of tumor growth and describes a condition in which the oxygen demand within an organism, cell, or tissue exceeds the available supply, typically described as < 10 mmHg O_2_ (6). Hypoxia is frequently found in solid tumors, including glioblastoma, due to rapid tumor growth, ultimately outstripping vascular supply and therefore, preventing O_2_ diffusion (6, 7). In gliomas, these hypoxic changes can be visualized on MR imaging, with high grade lesions displaying prominent ring-shaped contrast-enhancement with a hypointense center. Histological analysis of these regions often reveals highly anaplastic cells surrounding a necrotic tumor core, termed pseudopalisading necrosis (7, 8). The most important transcription factors in the cellular response to low pO_2_ are the hypoxia-inducible factors (HIFs). HIF-mediated signaling plays a role in vasculogenesis, tumor and cancer stem-like cell proliferation, and immunosuppression within the tumor microenvironment (TME). This family consists of a constitutively expressed β subunit (HIF1β), and at least three tightly regulated α subunits, HIF1α, HIF2α, and HIF3α. In normoxic conditions, the heterodimeric protein’s α subunit is rapidly degraded by the proteasome. Hypoxic conditions stabilize the α subunit and allow it to translocate to the nucleus, dimerize with the β subunit and induce transcription of hypoxia response genes (6, 7). In glioblastoma, it has been demonstrated that cells in the perivascular niche and necrotic areas upregulate the expression of HIF2α, and that this expression colocalizes with the stem cell markers CD133 and Olig2 (9, 10). Hypoxia increases growth and proliferation of glioma cells and glioma stem-cells, and strongly induces HIF2α, as well as stem genes. Ectopic expression of non-degradable HIF2α induced a stem-like phenotype in glioma cells and enhanced tumorigenicity *in vivo* (11). Hypoxia induced by the antibody bevacizumab that targets the vascular endothelial growth factor (VEGF), has also been shown to induce autophagy-related genes, resulting in a resistance mechanism to anti-VEGF therapy that could be abrogated by autophagy inhibition (12). In the past two decades, many basic investigations focusing on targeting hypoxia to increase the efficacy of VEGF inhibition were initiated, including inhibiting autophagy with chloroquine, or attempting to prevent HIF1α synthesis with mTOR inhibitors such as temsirolimus and everolimus (13).

**Figure 1:**
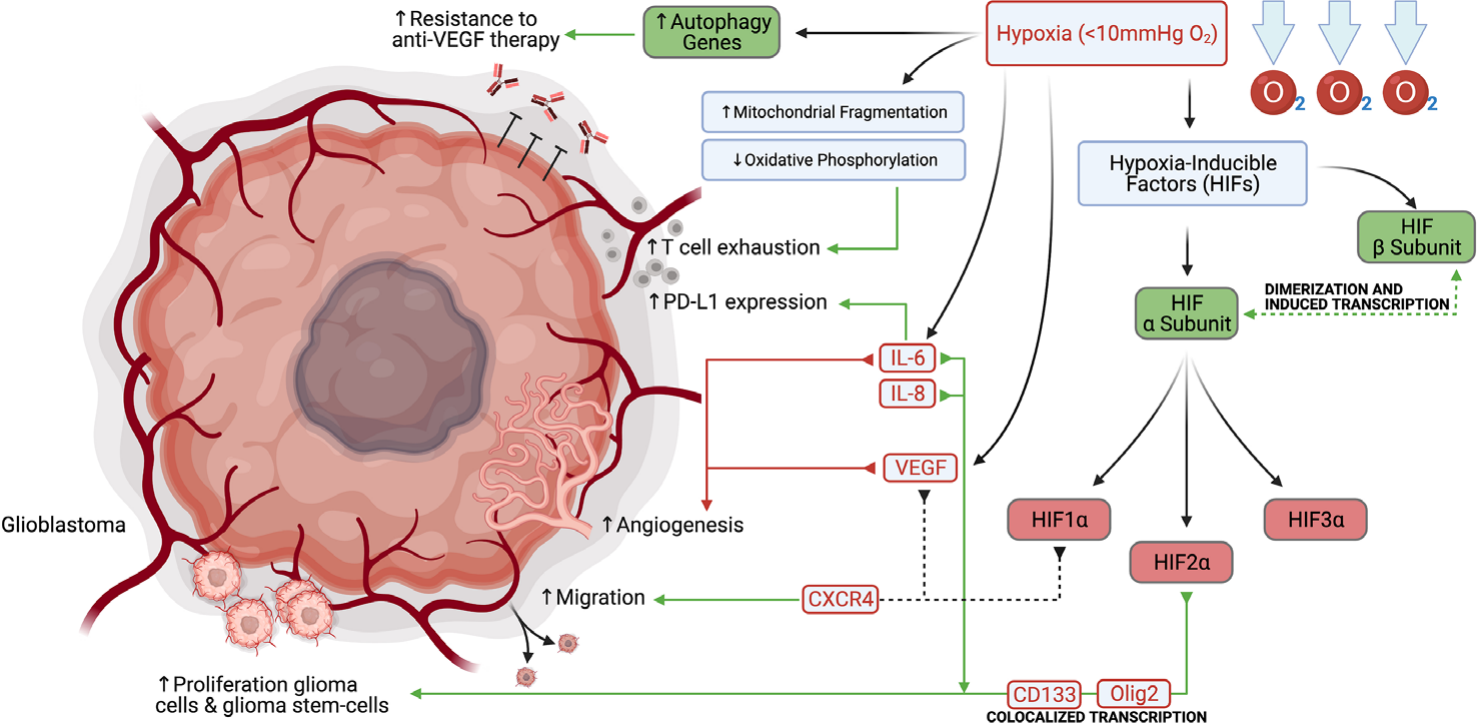
Hypoxia promotes glioblastoma and glioblastoma stem-cell proliferation, migration, angiogenesis, PD-L1 expression, and resistance to anti-VEGF therapy.

Hypoxic changes in the glioma microenvironment may also modulate key immune effector molecules (**Figure 1**). Hypoxia has been further shown to induce T cell exhaustion through mitochondrial fragmentation and decreased oxidative phosphorylation, among other mechanisms (14). Furthermore, under hypoxic conditions, glioma cells secrete interleukins IL-6 and IL-8, which serve as autocrine proliferative signals and localize to perinecrotic regions with many pseudopalisading glioblastoma cells (15, 16). IL-6 signaling also plays a role in maintaining the tumor stem cell niche and stimulating angiogenesis (15, 17). Finally, IL-6 has been shown to induce upregulation of the programmed death-ligand 1 (PD-L1) on tumor-infiltrating and circulating myeloid cells (18). Additionally, glioma cells and proliferating endothelial cells in the hypoxic perivascular niche respond to HIF1α and VEGF signaling by upregulation of the chemokine receptor CXCR4, allowing for increased migration (19).

A small-molecule HIF2α inhibitor PT2385, which was later improved to the second-generation inhibitor PT2977 (now known as ‘MK-6482’), has also been shown to block the transcription of HIF2α-responsive genes, including *VEGFA*, *CCND1* and the glucose transporter–encoding gene *SLC2A1*, and both molecules demonstrated on-target antitumor activity in mouse xenograft models of renal cell cancer (20). PT2385 showed promising preliminary promising activity in early phase development. This is also studied in glioblastoma and in a combination study with nivolumab (21). MK-6482 is nearly identical to PT2385, but with a more favourable pharmacokinetic profile and is also undergoing evaluation in early phase trials (20).

## Glycolysis

Glycolysis is the primary metabolic pathway that provides energy and involves the breakdown of glucose to form the high energy molecules ATP and NADH. The brain is an energy demanding organ with about 25% of the body’s glucose consumption being devoted to brain function (22). Despite the brain’s high energy demand, it has relatively low levels of glucose when compared to plasma (23). Glucose transporter 1 (GLUT1) is responsible for shuttling glucose into the brain, as well as driving it into cells (24). Neurons, oligodendrocytes, astrocytes, and tumor cells are especially dependent on glucose for survival and energy production (25, 26). Neurons additionally express Glucose transporter 3 (GLUT3), which is five-fold more efficient at transporting glucose than GLUT1 (24). The PI3k-Akt-mTOR pathway is primarily responsible for fulfilling the energy demands of transformed cells, neurons, and glia (27, 28).

Aerobic glycolysis (Warburg’s effect) is a hallmark of cancer and is a process through which cancer cells produce lactate after undergoing glucose-mediated oxidative phosphorylation (29). Like other tumors, glioblastoma highly expresses GLUT1 and its energy demands are greater than that of normal brain cells (30, 31). Transformed, neuronal, and glial cells have high energy demands in a low glucose environment, and act as a sink that depletes glucose which limits immune cell anti-tumor effector functions. Gliomas can further recruit and maintain immunosuppressive immune populations such as pro-tumor mononuclear phagocytes which also undergo glycolysis and deplete available glucose, among other nutrients including L-arginine and L-cysteine, from the tumor microenvironment (32, 33). Blockage of the Akt-mTOR pathway *via* administration of Akt inhibitors in low glucose environments has been shown to inhibit growth of glioma cells (34). Additionally, high expression of a glycolysis related gene signature was associated with cancer progression, adhesion, proliferation, angiogenesis, and drug resistance, further demonstrating the important role of glycolysis in glioblastoma (35).

**Figure 2:**
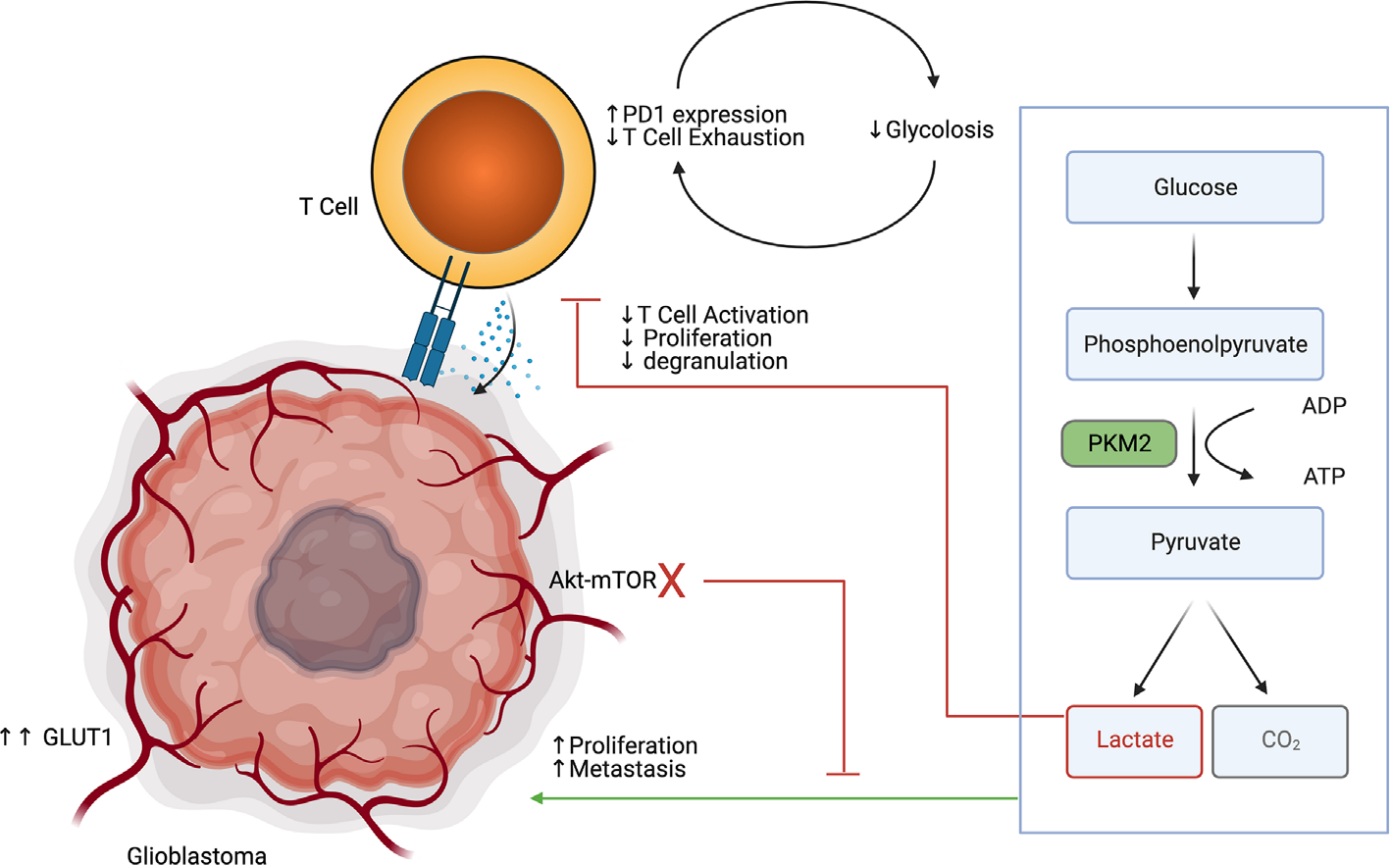
Glioblastoma glycolysis drives tumor progression while inhibiting T cell glycolysis, activation, proliferation and degranulation.

Immune cells, and in particular effector T cells, are dependent on glycolysis to support their proliferation and effector functions (36) (**Figure 2**). TCR signaling in T cells results in PI3k-Akt-mTOR signaling, which further increases glucose requirements (37). Low glucose availability is a known driver of the exhaustion phenotype in T cells (38). Recent research suggests that exhausted T cells exist on a continuum from a precursor exhausted state, that are responsive to checkpoint blockade, to a terminally exhausted state, which are refractory to checkpoint blockade therapy (39). Precursor exhausted T cells have been shown to have reduced expression of glycolysis related genes in relation to naïve and effector T cells (40). Interestingly the PD1/PDL1 receptor ligand pair has divergent functions in T cells and the tumor. Ligation of PD1 on T cells reduces glucose uptake, where increased expression of PDL1 on tumor cells improves tumor glycolysis (41–43). Reinforcing exhaustion in T cells *via* inhibitory signals and reducing glucose uptake is a synergistic way that the tumor cells maintain the immunosuppressive microenvironment. Furthermore, lactate accumulation in the glioblastoma TME is in of itself a potent immunosuppressive agent. Lactate has been shown to polarize macrophages towards an M2 phenotype, impair lymphocyte proliferation, activation, and degranulation (44). Broad pharmaceutical inhibition of glycolysis may not result in a net anti-tumor effect, as it has pro-tumor effects in glioma cells, but is also important for anti-tumor effects in T cells. Drugs that disrupt glycolysis preferentially in the tumor or bolster glucose uptake specifically in T cells would be attractive methods of leveraging metabolism to provide an anti-tumor effect.

## Tryptophan Metabolism

The tryptophan catabolism is among the most characterized immunometabolic pathways in glioblastoma since it contributes to both tumor progression and immune evasion. Tryptophan, the least abundant amino acid, can be incorporated into proteins, modified to produce serotonin, or metabolized to produce kynurenines (Kyn). Briefly, tryptophan is metabolized by indoleamine 2,3-dioxygenase (IDO)1/2 and tryptophan 2,3-dioxygenase (TDO), another important enzyme of the kynurenine pathway, to produce N‐formyl kynurenine, which is converted to kynurenine by arylformamidase (AFMID). Kynurenine is further metabolized through various pathways to produce metabolites including kynurenic acid, anthranilic acid, 3‐hydroxykynurenine, xanthurenic acid, quinolinic acid, picolinic acid, and nicotinamide‐adenine‐mononucleotide (45). Although difficult to control each of these metabolites’ pathways individually, it has been possible to inhibit their production through upstream IDO1/2 and TDO inhibition. IDO1/2 expression are typically found in peripheral tissues, while TDO expression is associated with hepatic tryptophan metabolism. Although recent evidence suggests that there may be value in specifically targeting TDO in the context of glioblastoma, tryptophan metabolism in glioblastoma has primarily been explored in the setting of IDO1/2.

Kynurenines and quinolinic acid have been previously described to be able to drive neoplastic proliferation through Wnt/β‐catenin signaling (46). Kynurenines further influence tumorigenesis in glioblastoma by modulating DNA repair enzyme, polymerase kappa, thereby preventing DNA damage and allowing genomic instability to propagate leading to tumor heterogeneity (47). Kynurenines and quinolinic acid may also promote cell proliferation in a fibroblast growth factor‐1 (FGF‐1) dependent manner (48). Lastly, nicotinamide‐adenine‐mononucleotide can be converted to NAD+, which confers tumor cells’ resistance to oxidative stress. Although the mechanism remains largely unclear, IDO expression may play a role in tumor angiogenesis and metastasis through control of IFNg and IL-6 (49). Mondal et al. found that *in vivo* IDO inhibition reduced metastasis and neovascularization (50). In patients with glioblastoma, IDO expression was strongly associated with shortened overall survival (51).

**Figure 3:**
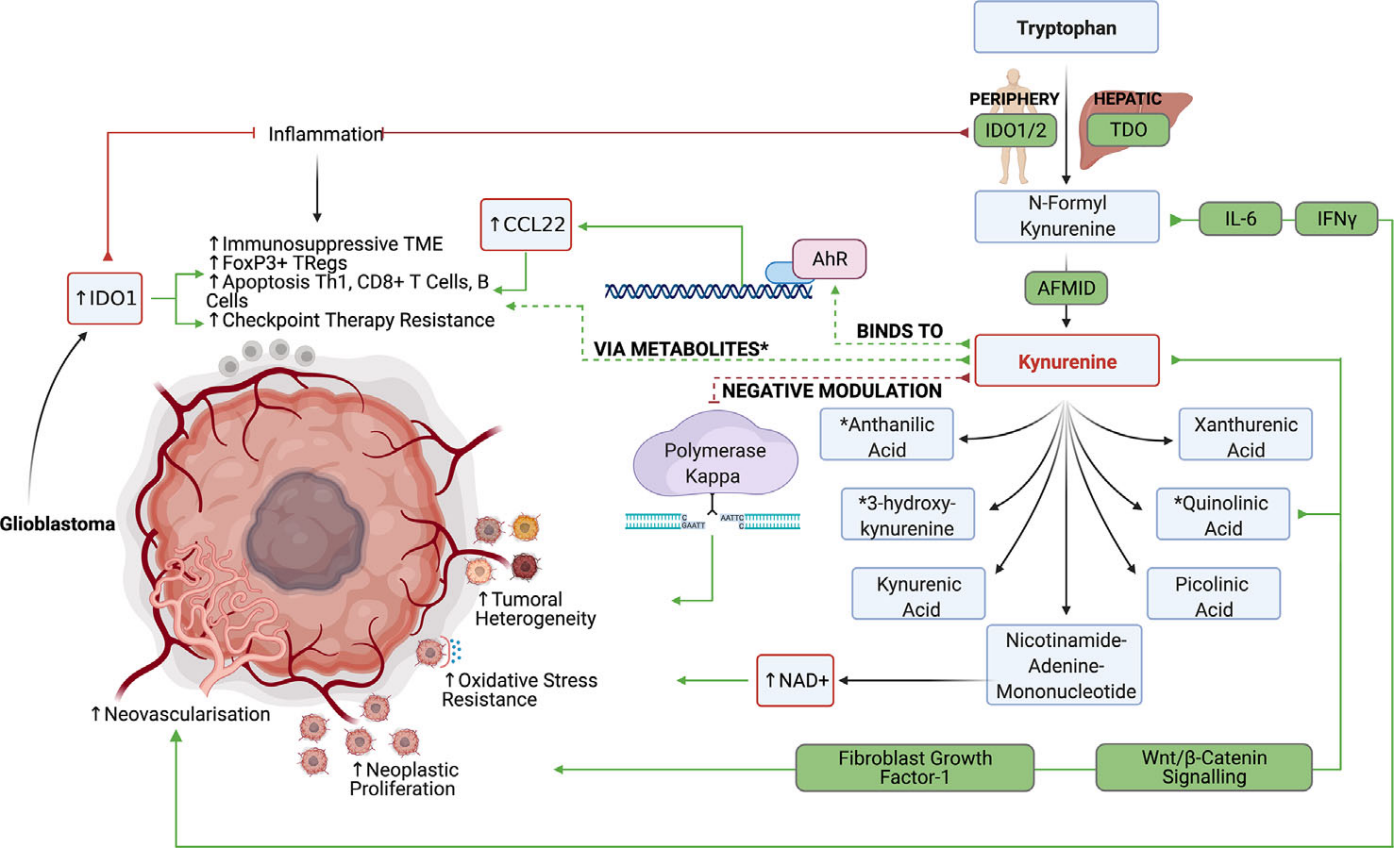
The IDO1/2 and TDO pathway allow the production of various tryptophan metabolites, which suppress anti-glioblastoma lymphocyte responses while promoting tumor growth.

IDO1/2 mediated depletion of tryptophan was initially thought of as an ancient innate immune mechanism to prevent the growth of microorganisms while reducing inflammation and autoimmunity in areas such as the brain (52). In fact, IDO1/2 expression is significantly increased in inflammatory tissues due to IFNg, TGF-β, and PGE2 signaling to potently inhibit active inflammation (**Figure 3**). Wainwright et al. were among the first to demonstrate that glioblastoma cells significantly upregulated the expression of IDO1 and suggested that IDO1 may also contribute to tumor progression by promoting an immunosuppressive phenotype (53).

In both clinical studies and preclinical murine glioma models, tumors with high expression levels of IDO1 were infiltrated with more FoxP3+ regulatory T cells (53). It has been suggested that tryptophan metabolism can induce Treg differentiation based on the kynurenines’ ability to bind to cytosolic ligand-activated transcription factor AhR (54). Kynurenine-driven AhR activation also induces the production of CCL22 to recruit Tregs into the glioblastoma TME (55). In addition to inducing suppressive T cell populations, tryptophan metabolism may blunt antitumor CD4 and CD8 responses through various mechanisms. Opitz et al. found that TDO derived kynurenines reduced the proliferation of both CD4+ and CD8+ T cells and reduced LCA+ CD8+ T cell infiltration in human gliomas with high TDO expression (56). Furthermore, depletion of tryptophan in the TME leads to an accumulation of unbound tryptophan–tRNA in T cells which activates the GCN2 mediated stress response and inhibits RNA transcription and protein synthesis in T cells leading to cell cycle arrest and apoptosis (57). Although currently contested, some evidence suggests that tryptophan deprivation may also inhibit the mTOR pathway in T cells to inhibit effector T cell functions. Additionally, metabolites like quinolinic acid, 3-hydoxyanthranilic acid, and 3‐hydroxykynurenine have been shown to induce apoptosis in Th1 helper cells, CD8+ Effector T cells, B cells, while sparing immunosuppressive Th2 helper cells.

IDO inhibitors such as 1-L-MT, IDO-IN-2, Navoximod (GDC-0919), IDO-IN-1, Linrodostat, coptisine chloride, PF-06840003, and TDO inhibitors such as 680C91 have recently been developed (58). While IDO inhibitors such as navoximod did not improve antitumor responses in preclinical glioblastoma models, Kesarwani et al. found that navoximod synergistically improved antitumor responses when combined with RT and immune checkpoint blockade (59). Hanihara et al. and Li et al. similarly found that while 1-L-MT did not improve antitumor immunity on its own, 1-L-MT significantly synergized with temozolomide administration and radiation therapy (60, 61). Wainwright et al. found that IDO inhibitors particularly synergized with PD1 and CTLA4 blockade in the mice (62, 63). Interestingly, advanced age is associated with an increase of brain IDO expression and this is not reversed by IDO enzyme inhibitor treatment (64). It remains to be seen if targeting IDO will translate into clinical benefit in cancer and in gliomas.

## Arginine Metabolism

Arginine is yet another amino acid substrate that is actively metabolized by tumor cell to promote tumor progression and immunosuppression. L-arginine is critical in the urea cycle and is a modulator of immune function and tumor metabolism. L-arginine is utilized as a substrate for both Arginase 1 (ARG1) and cytokine inducible nitric oxide synthase (iNOS). ARG1 converts L-arginine to urea and ornithine, which is further utilized in the urea cycle. iNOS converts L-arginine to citrulline and nitric oxide (NO), which is important for directing anti-tumor functions in immune cells (65).

**Figure 4:**
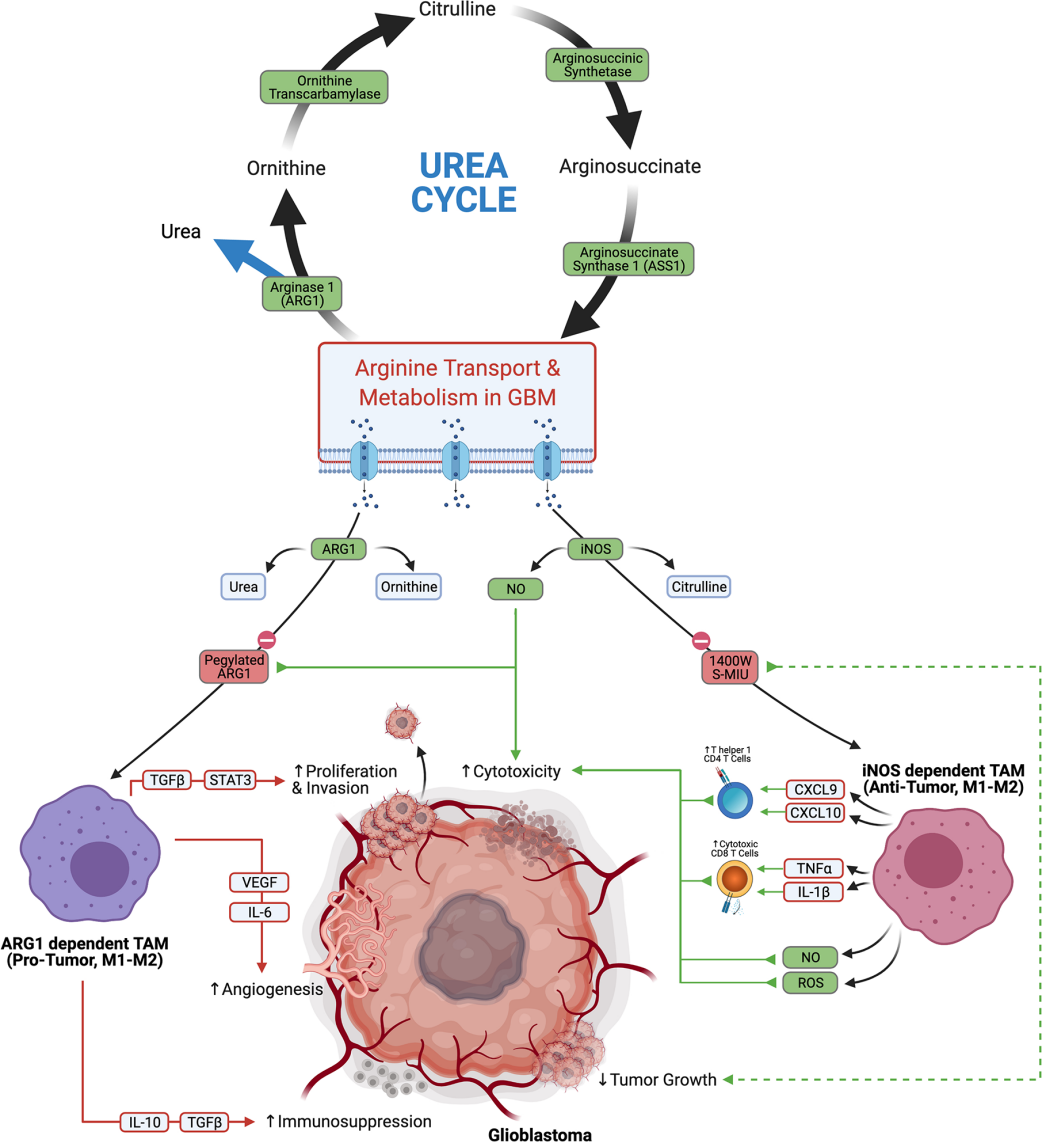
Glioblastoma arginine metabolism *via* iNOS or Arg1 polarizes the tumor associated macrophages towards anti-tumor tumor or pro-tumor phenotypes, respectively.

Depletion of arginine has been identified as a successful treatment strategy in cancers that are deficient in aspects of arginine metabolism and are reliant on exogenous sources (**Figure 4**). This approach has been successful in Leukemia, where transformed cells were found to be deficient in asparagine synthase and were not capable of producing asparagine. This left the tumors vulnerable to treatment with L-asparaginase which depleted asparagine (66). Similarly the function arginosuccinate synthase 1 (ASS1) is defective in some tumors, which makes them dependent on exogenous arginine (66). In glioblastoma, there seems to be an abundance of arginine transporters, which is evidenced by a notable accumulation of byproducts of arginine metabolism (67, 68). This suggests that arginine metabolism is functional, and may be sensitive to targeted depletion. A recent study that utilized a pegylated recombinant human ARG1, depleted arginine, and induced cytotoxicity in glioma cells (69). Similarly, selective iNOS inhibitors 1400W and S-MIU have recently been shown to reduce tumor growth in a EGFRvIII mutant overexpressing U87 glioblastoma model (70). How arginine fosters immunosuppression in the TME is also an area of research that seeks to elucidate the tumor promoting effects of arginine metabolism.

Macrophages are a large component of the TME, constituting up to 30% of the tumor by weight (71, 72), and the divergent functions of arginine metabolism are best appreciated in this immune cell subset. Macrophages are a highly plastic cell type, which can adopt either pro- or anti-tumor function depending on their environmental cues. Macrophage polarization has traditionally been thought of to exist on a continuum from M1 to M2 phenotypes which confer inflammatory/anti-tumor phenotypes and repair/pro-tumor phenotypes, respectively. Recent research suggests that the M1-M2 dichotomy is likely an oversimplification (73), which is underscored by the numerous reports that tumor associated macrophages (TAMs) in glioblastoma express a mixture of M1 and M2 related genes (74, 75). TAMs which primarily metabolize arginine *via* iNOS are considered more anti-tumor. iNOS dependent anti-tumor TAMs skew the TME towards cytotoxicity through stimulating Th1 responses *via* secretion of CXCL9 and CXCL10, inducing cytotoxic CD8s through TNFα and IL1β, and direct killing of tumor cells through nitric oxide (NO) and Reactive Oxygen Species (ROS). Conversely TAMs which metabolize arginine primarily through ARG1 are thought to have more pro-tumor activity. ARG1 dependent pro-tumor TAMs stimulate angiogenesis through VEGF and IL6, promote invasion and proliferation *via* TGFβ and STAT3, and support immunosuppression through IL-10 and TGFβ (65). TAMs in glioblastoma are considered to be pro-tumor overall, and their accumulation correlates with worse prognostic outcomes (76). Due to the highly plastic nature of TAMs and their abundance in the glioblastoma TME, they are an attractive target for repolarization from a pro-tumor to an anti-tumor phenotype. Finding ways to selectively shift arginine metabolism in TAMs towards iNOS presents an interesting treatment modality that could potentially skew a large portion of the TME to an overall anti-tumor effect.

## 2-Hydroxyglutarate Metabolism

2HG production represents a unique immune-metabolomic pathway found in many cancer cells, including low-grade gliomas and secondary glioblastoma. Within low-grade gliomas and secondary glioblastoma, 2HG is often produced due to mutations in the catalytic domains of isocitrate dehydrogenase isoform 1 (IDH1) and isocitrate dehydrogenase isoform 2 (IDH2). The most common mutations in IDH1 include R132H, R132C, R132L, R132S, and R100Q, while the most common mutations in IDH2 include R140Q, R140G, R140W, R140L, R172K, R172G, R172M, R172Q, R172T, R172S (77). While IDH1 is found in the cytoplasm of cells, IDH2 is found in the mitochondrial matrix. Despite their differences in cellular sub-localization, both wildtype IDH1 and wildtype IDH2 catalyze the decarboxylation of isocitrate using NADP^+^ and Mg^2+^ as cofactors and produce α-Ketoglutaric acid and CO_2_ (78). Wildtype IDH1 and wildtype IDH2 are normally also able to catalyze the reverse reaction by reducing α-Ketoglutaric acid into isocitrate using NADPH as a cofactor. α-Ketoglutaric acid also acts as a substrate for an alternative reduction reaction that incompletely reduces α-Ketoglutaric acid into 2-Hydroxyglutarate instead of isocitrate in an NADPH driven manner. Somatic missense mutations of arginine in IDH1 and IDH2 lead to impaired oxidative carboxylation and favor the incomplete reduction of α-Ketoglutaric acid into 2-Hydroxyglutarate (79). While 2HG is produced by other enzymes including hydroxyacid-oxoacid transhydrogenase (80), human phosphoglycerate dehydrogenase (81), lactate dehydrogenase (82), and l-malate dehydrogenase (83), it is believed that IDH1/2 mutations are almost exclusively what drive 2HG overaccumulation in low-grade glioma and secondary glioblastoma. In grade II/III gliomas carrying IDH1/2 mutations, 2HG concentrations have been found between 1 and ∼30 m*M* (77). The overaccumulation of 2HG can both promote gliomagenesis while inhibiting anti-tumor immunity.

The oncogenic process can be mediated through epigenetic regulation, 2HG, inhibition of DNA repair enzymes, promotion of autophagy, and promotion of invasiveness. 2HG exerts control over cellular epigenetics by favoring the hypermethylation of various genes by inhibiting α-Ketoglutaric acid-dependent dioxygenases such as Tet methylcytosine dioxygenases (TETs) (84). Koivunen et al. demonstrated that TET2, in particular, was inhibited by 2HG (85). TET2 inhibition was demonstrated to decrease tumor cell differentiation and promote tumorigenesis in the setting of glioblastoma by Garcia et al. (86) Perhaps most notably, 2HG was shown to increase methylation of histone lysines and c-Myc binding at the promoter of the telomerase reverse transcriptase (TERT) gene encouraging tumor transformation and immortalization (87). 2HG was also shown by Chen et al. to inhibit the AlkB family of DNA repair enzymes such as ALKBH2 and ALKBH3 (88). 2HG further promotes autophagy and cell survival by indirectly controlling mTORC1 and mTORC2 signaling. 2HG does this by activity inhibiting KDM4A, which allows DEPTOR to activate the mTORC1/2 pathway (78). 2HG may also have a role in destabilizing the basement membrane of glioblastoma cells through the inhibition of collagen stabilizing enzymes such as PLOD1, PLOD3, P4HA1, and PHA3 (79). In addition to IDH1/2 mutations helping produce 2HG, these mutations also favor the consumption of NADPH instead of their production. The depletion of NADPH helps induce cellular dysregulation and impairs cellular defense against reactive oxygen species (77).

**Figure 5:**
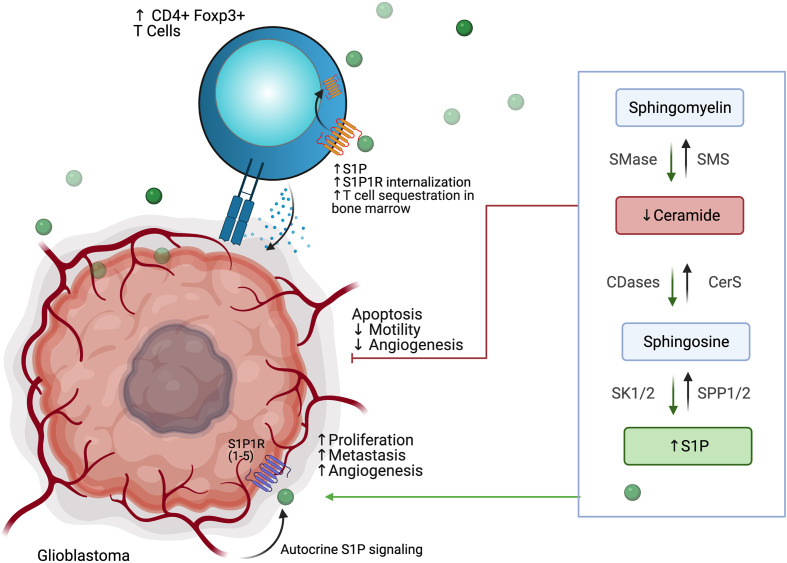
2 HG produced by IDH1/2 mutant secondary glioblastoma promotes tumor survival while impairing T cell activation and degranulation.

While 2HG has been shown to influence tumor growth, 2HG has also been demonstrated to modulate anti-tumor immunity (**Figure 5**). Bunse et al. demonstrated that 2HG produced by tumors can be transported into immune cells *via* SLC13A3, which generally impairs immune function (89). Within T cells, 2HG was shown to inhibit T cell activity by inhibiting enzymes such as ornithine decarboxylase, transcription factors such as NF-κB p65, and the NFAT pathway through NFATC1 (89). It was demonstrated that T-cells treated with 2HG producing astrocytes demonstrated decreased production of IFN-γ and IL-2 upon activation (89). 2HG also directly inhibits T cell activation by inhibiting the steps that lead to calcium influx, such as early ATP-dependent TCR signaling events, c-Jun N-terminal kinase (JNK), and PLC-γ1(Y783) phosphorylation (89). Of note, the effect of 2HG on the suppression of T-cell activation was most prominent in CD4+ T cells. 2HG was also found by Kohanbasch *et al.* to reduce the expression of STAT1 in DC cells, thereby inhibiting the secretion of CXCL10 in the glioblastoma TME (90). This represents yet another mechanism by which 2HG may suppress T cell activity. Interestingly, IDH1/2 mutant tumors are generally infiltrated by T cells expressing less PD-1 than those T cells found in IDH1/2 wildtype tumors (89). This may be due to 2HG inhibiting NFAT translocation, which is necessary for inducing PD-1 expression. IDH1/2 mutant tumors are also generally infiltrated by less immunosuppressive M2 Macrophages (91). Amankulor et al. suggest that the decreased immunosuppressive cell infiltration in IDH1/2 mutant tumors may play a role in controlling the growth of secondary glioblastoma (92).

Given the immunometabolic importance of IDH1/2 mutations, IDH mutation-specific inhibitors have been developed. IDH1 mutations have been targeted through molecules such as Ivosidenib, BAY-1436032, and AG-5198 (93). IDH2 mutations have been targeted through molecules such as Enasidenib, AGI-6780, and GI-6780 (93). IDH1 and IDH2 mutations have been co-targeted though molecules such as AG-881, which is currently being evaluated in the phase 3 INDIGO trial (NCT04164901) in patients with residual or recurrent Grade 2 glioma with an IDH1 or IDH2 mutation (93). Since IDH mutations may help anti-tumor immunity by decreasing PD1 expression and decreasing immunosuppressive cell infiltration, there may rationale to combine IDH mutation inhibitors with other immunostimulatory therapies such as checkpoint blockade. Bunse et al. demonstrated that BAY1436032 and PD-1 therapy increased overall survival in a murine IDH1 R132H model (89). Similarly, Kadiyala et al. found that 2HG inhibition, IR treatment, temozolomide, and anti-PDL1 administration led to complete tumor regression in 60% of mice bearing IDH1 mutant gliomas (94). In addition to small molecule inhibition, Platten et al. recently demonstrated that the IDH1 R132H mutant pathway could also be targeted by a peptide vaccine approach in newly diagnosed gliomas (95). While IDH inhibitors and vaccines represent one of the remarkable success stories in low grade IDH mutated gliomas, they are unfortunately not a therapeutic option in IDH wild type glioblastoma.

## Adenosine Metabolism

In normal physiology, adenosine and ATP are found in the cytosol of tissues, while these metabolites’ extracellular levels are rarely observable (96). In certain pathologies such as gliomagenesis, intracellular adenosine can be secreted *via* bidirectional equilibrating nucleoside transporters (97), and ATP is released extracellularly *via* plasma membrane disruption or ATP efflux induced by hypoxia or inflammation and mediated by ABC transporters, anion channels, connexins, pannexins, and receptors like P2X7R (98). Once in the extracellular environment, ATP is degraded by enzymes such as CD73 and CD39 to produce adenosine. While the CD73 and CD39 mediated pathway of adenosine production are the most relevant to glioblastoma, extracellular adenosine may also be generated *via* ecto-phosphodiesterase/pyrophosphatase family proteins, nicotinamide adenine dinucleotide glycohydrolases, prostatic acid phosphatase, and alkaline phosphatase (99, 100). Extracellular adenosine is regulated either by cellular uptake or extracellular adenosine deaminase enzymes. The extracellular adenosine that remains can signal through high-affinity A2a and low-affinity A2b receptors expressed on tumors, tumor-associated cells, and immune cells. Blocking this adenosine signaling represents an intriguing target to modulating anti-tumor responses.

The adenosine metabolism pathway is of particular importance in glioblastoma and low-grade gliomas. Ott et al. recently found that in patients with gliomas, the A2aR/CD73/CD39 pathway was most frequently expressed (101). In the hypoxic glioblastoma TME setting, there is an increased expression of HIF1a in tumor tissue leading to increased expression of CD39 and CD73 on tumor cells, immune cells, stromal cells, and endothelial cell, leading to increased extracellular adenosine. This extracellular adenosine can signal through adenosine receptors to improve tumor survival, stimulate tumor cell proliferation, and induce tumor cell invasion and angiogenesis. Adenosine can improve tumor cell survival through the AKT and ERK pathways, inhibiting caspase pathway activation, upregulation of Bcl2 family antiapoptotic genes and downregulation of P53 (102, 103). Adenosine has been shown to induce tumor cell proliferation through various pathways including but not limited to AKT, ERK, JNK, and P38, ERa, and upregulation of cyclin proteins (104–106). Adenosine has been shown to increase the expression of matrix metalloproteases (MMPs) and FXYD5, which disrupt the tumor’s ECM and drive invasiveness by reducing cell adhesion, respectively (107). Lastly, adenosine signaling can help drive angiogenesis by inducing increased VEGF, IL8, angiopoietin 2, and erythropoietin (100).

**Figure 6:**
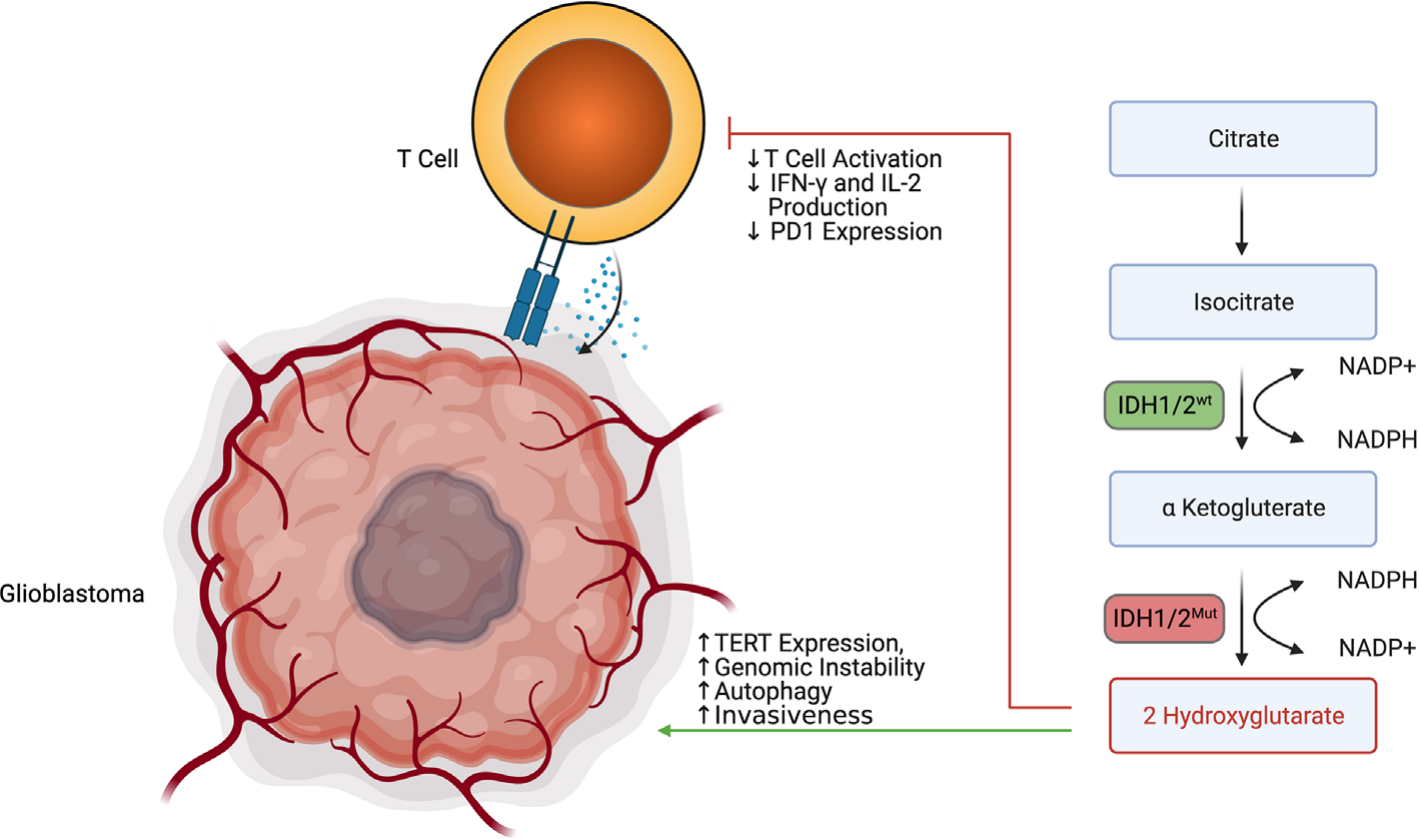
Glioblastoma cells control the amount of extracellular adenosine in the glioblastoma immune microenvironment and use adenosine to decrease T cell degranulation and increase T cell exhaustion.

In addition to regulating tumor growth, adenosine signaling has a multifaceted role in controlling anti-tumor immunity (**Figure 6**). While extracellular ATP functions as danger associated molecular pattern (DAMP) that can stimulate both innate and adaptive immunity, extracellular adenosine serves to dampen the immune system (108). Within T cells, adenosine signaling through receptors such as A2aR inhibits MAP kinase, protein kinase C, NFkB, and NFAT pathways and inhibit proximal TCR signaling (109, 110). T cells treated with adenosine demonstrated decreased IL-2, TNFa, and INFg production, decreased CD28 expression, and increased expression of PD1, CTLA4, and LAG3 (108, 111, 112). Adenosine signaling was also shown to help generate Tregs by increasing the expression of FoxP3 in CD4 T cells (113). These Tregs were found to upregulate CD39 and CD73, creating a positive feedback loop in the TME (113). Adenosine signaling similarly blunted NK cell target cell killing, proliferation, and the production of IFNg and TNFa (114–116). DC cells treated with adenosine exhibited decreased expression of TNFa and IL12 while increasing their expression of immunosuppressive factors including IL-5, IL-6, IL-10, TGFβ, arginase, and IDO, and PD-L2 (117, 118). In macrophages, adenosine signaling *via* A2aR and A2bR were shown to induce M2 polarization (119) and blunted the secretion of neutrophil chemoattractants (111). Adenosine signaling also directly impaired neutrophil function finding their ability to adhere, degranulate, phagocytose, and produce TNFa and superoxide (120).

Sitkovsky et al. were one of the first to demonstrate enhanced anti-tumor immunity in A2aR knockout mice and demonstrated that A2aR could be inhibited using pharmacologic blockade (121, 122). In preclinical models, the immunosuppressive effect of adenosine signaling has been blunted using anti-CD73 and anti-CD39 blockade (123). In multiple murine tumor models, A2aR inhibition synergized with anti-PD1, anti-TIM3, and anti-CTLA4 antibodies to improve survival and reduce tumor metastasis (124–126). Inhibitors of this pathway such as Istradefylline, SCH-442416, Preladenant, BAY-545, Ciforadenant (CPI-444), Imaradenant (AZD4635), SCH58261, AB928 and AB680 have emerged and many of them are in clinical development (123). Targeting the adenosine signaling pathway in glioblastoma remains an active area of research.

## Altered Phospholipid Metabolism

Sphingolipids are important structural components of the cell membrane that play a role in membrane fluidity and integrity. Many sphingolipids are also highly bioactive and play roles in a variety of cellular processes. Sphingosine, the first discovered sphingolipid, is induced by cellular stressors, including chemotherapy and radiation, and functions in cytoskeletal reorganization, cell cycle regulation, senescence and apoptosis. Since then, many other sphingomyelins have been identified, including ceramide, a molecule involved in regulation of apoptosis and is believed to be the central hub of sphingolipid metabolism, as well as sphingosine-1-phosphate, or S1P, which has roles in promoting survival, migration, and inflammation (127, 128).

Sphingolipid synthesis occurs *de novo via* condensation of serine and palmitate to 3-keto-dyhydrosphingosine, which *via* several intermediate steps involving ceramide synthases (CERS1-6) is converted to ceramide, a molecule well-identified as a pro-apoptotic signal (128, 129). Ceramide consists of an 18-carbon sphingosine long-chain base that contains an amide-linkage to a fatty acyl chain of variable carbon number; synthesis by CERS1-6 is the rate-limiting step of *de novo* ceramide synthesis and each enzyme is responsible for ceramides of specific fatty acyl chain length. Ceramides can also be synthesized *via* salvage following breakdown of complex sphingolipids such as sphingomyelins, *via* the subcellularly-localized sphingomyelinases (acid, neutral, and alkaline SMases), and cerebrosides, *via* glucosylceramidase and galactosylceramidase. Ceramide breakdown *via* ceramidases leads to sphingosine formation, which may be recycled or phosphorylated by the sphingosine kinases SK1 and Sk2 to form S1P. S1P is a ligand for the five G-protein coupled receptors S1PR1-5 and is normally rapidly metabolized *via* S1P phosphatase (SGPP) and S1P lyase 1 (SPL). Activation of S1PRs results in cellular proliferation and further production of S1P to promote cell motility and survival. This tight linkage of interconnected pathways for the rapid synthesis and breakdown of ceramide (pro-apoptotic) and S1P (pro-survival) has given rise to the “sphingolipid rheostat” model, in which the balance of these two biomolecules plays an important and potentially targetable role in normal cellular function and oncogenesis (127–131).

**Figure 7:**
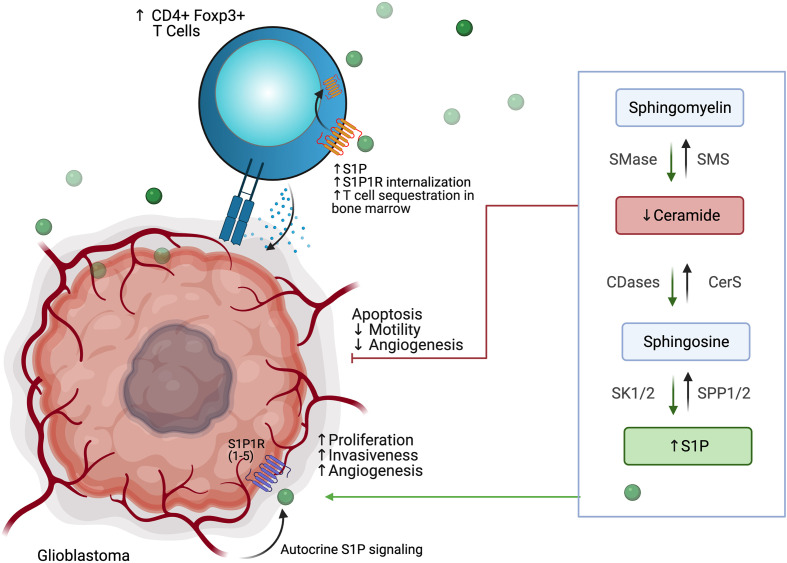
Glioblastoma cells use altered phospholipid metabolism to induce tumor proliferation, invasiveness, and angiogenesis while sequestering T cell populations away from the tumor microenvironment.

Derangement of the sphingolipid rheostat is implicated in the pathogenesis of glioblastoma (**Figure 7**). Analysis of human glioma tissue revealed significantly lower ceramide levels in high grade tumors relative lower grade tumors, and relative to peritumoral brain tissue (132). This difference was most dramatic for the C18 ceramide. Likewise, S1P levels in glioma tissues were higher than in normal gray matter; glioma stem-like cells have also been shown to secrete S1P as an autocrine, resulting in proliferation and increased expression stemness markers (132, 133). Taken together, this S1P/ceramide shift represents a common, targetable feature of malignancy even with regard for the heterogeneity displayed within and between glioblastomas. In gliomas, this shift is likely due to multiple alterations in sphingolipid synthesis, including increased expression of SK1 (134–136), deletion of chromosomal regions containing SPL (137), and downregulation of S1P phosphatase 2 (138), resulting in increased S1P levels; as well as inhibition of ceramide synthase (139) and increased expression of ceramidases (134). The activity of SMases in glioblastoma is still being elucidated, but it has been shown that acid SMase may sensitize glioma cells to chemotherapy and radiation by increasing metabolism of sphingomyelin to ceramide and consequent apoptosis in the context of p53-deficiency; conversely, neutral SMase may be involved in increasing ceramide production in p53-wildtype cells (129). Increased understanding of the proapoptotic role of SMases in glioblastoma may yield new therapeutic targets.

Interactions of glioblastoma with surrounding neuronal, glial, and immune cells in the TME are continuing to be appreciated. As discussed above, S1P plays an autocrine role in gliomas, and has been shown to be constitutively secreted in rodent glioma cells and human glioblastoma cell lines, likely due to SK1 activity (134, 140, 141). Increased SK1 activity, in turn, has been shown to be induced by microenvironmental IL-1 and HIF-2α activity (142, 143). S1P is also capable of acting as a chemoattractant for innate and adaptive immune cells (144, 145). Increased glioblastoma-derived S1P may thus promote formation of TAMs. TAMs, in turn, may also contribute S1P to the TME and increase SK1 activity (130). TAMs also produce NO, which has been shown to decrease acid SMase activity in glioma cells, resulting in therapeutic resistance (146). Despite the breakdown of the blood-brain barrier (BBB) in glioblastoma, there is often a paucity of T-cells in the TME or within the tumor (so-called tumor-infiltrating lymphocytes, or TILs). Those cells that are present are often the CD4+ CD25+ FoxP3+ population of immunosuppressive regulatory T-cells, termed T_regs_ (147). In addition to their role in apoptosis, global alterations of SMase expression modulate differentiation of T cell populations, with acid SMase activation linked to increased numbers of CD4+ Th1 and Th17 cells, while mice deficient in acid SMase exhibit increased T_regs_ (148, 149). Recently, it has been shown that glioblastomas may cause sequestration of T cells in bone marrow *via* T-cell internalization of S1PR1, and enforced expression of S1PR1 in combination with T-cell activation *via* 4-1BB agonism can increase survival *in vivo* (144). 4-1BB agonism has also been shown to rescue the poor efficacy of PD-1 blockade in glioblastoma *in vivo*; translation of these results to clinical trials is greatly anticipated (150). Blockade of S1PR1 is commonly employed using the sphingosine analog fingolimod to reduce immune trafficking in multiple sclerosis; fingolimod may also inhibit ceramide synthases, SK1, and SPL and was proposed as a possible therapeutic for glioblastoma (144, 151). A small trial of fingolimod was initiated with the aim of assessing whether sequestration of lymphocytes *via* S1PR1 antagonism could reduce post-chemoradiation lymphopenia in glioblastoma patients, but results have not yet been published (NCT02490930). Interestingly, Baeyens et al. recently found that monocytes in the lymph node may also produce S1P and influence T cell differentiation and T cell residence time in the lymph node (152). Their work suggests an alternative mechanism by which drugs that target S1P signaling can influence the glioblastoma TME and immune populations.

Additionally, an emerging mediator of aberrant phospholipid metabolism in glioblastoma is polymerase I and transcript release factor (PTRF), also known as Cavin1. PTRF was originally discovered to be involved in dissociation of RNA polymerase I-rRNA-DNA ternary complexes during transcription (153, 154). Through its colocalization with caveolin1 (Cav1) on the plasma membrane, it has been subsequently identified as essential for the formation of caveolae, cell-membrane infoldings that are 50-100 nm in diameter and function in cell signaling, lipid metabolism, and endocytosis (155, 156). Indeed, mutations in PTRF cause congenital generalized lipodystrophies in humans, providing further evidence for its role in lipid metabolism (157, 158). PTRF has also been shown to play a role in oncogenesis, as reduced PTRF expression in prostate and lung cancer is associated with progressive disease (45).

There is increasing evidence of a role for PTRF in the growth and progression of glioblastoma. PTRF has been shown to be upregulated in chemoresistant glioma cells and in human tumor tissues, with increasing PTRF expression correlating glioma grade and with tumor recurrence (155, 159). Huang et al. showed that EGFRvIII, an EGFR mutant with constitutively active tyrosine kinase activity present in ~25% of glioblastoma patients, drove PTRF upregulation (159). Blockade of PI3K and AKT reduced PTRF expression, showing a role for PTRF in EGFR-driven gliomagenesis even in the absence of the EGFRvIII mutation. This overexpression of PTRF in gliomas results in increased secretion of exosomes, cell growth, and aberrant methylation (159).

Interestingly, *in silico* analyses have suggested that PTRF expression is negatively correlated with the presence of cytotoxic lymphocytes intratumorally (160). Yi et al. recently showed that overexpression of PTRF in primary glioblastoma cells results in accumulation of lysophosphatidylcholine (LPC) species and decreased phosphatidylcholine (PC), resulting in increased membrane fluidity, endocytosis, and levels of the protein cytoplasmic phospholipase A2 (cPLA2), which provides fatty acids for mitochondrial fatty acid oxidation. *In vivo*, PTRF overexpression resulted in increased tumor growth and shorter survival. The authors found that intratumoral interferon gamma (IFN-γ) and granzyme B (GzmB) were decreased, with decreased numbers of CD8+ TILs, providing evidence for the role of abnormal phospholipid synthesis in glioblastoma immunosuppression. Strikingly, inhibition of cPLA2 restored IFN-γ and GzmB levels and resulted in increased TIL accumulation (161). Future investigations of cPLA2 in combination with existing immune activating therapies such as checkpoint blockade or CAR-T cells are warranted.

## Discussion

Several metabolic pathways are implicated in maintaining immunosuppression and glioblastoma outgrowth in the TME. These aspects have the potential to be exploited therapeutically but also for the development of diagnostic tools, including imaging tools such as MR spectroscopy for 2HG (162), hyperpolarized [1-13C] lactate (163), intratumoral acidity using pH-weighted amine chemical exchange saturation transfer (CEST) MRI (164) and amino acid PET tracers like 18F-fluoroethyltyrosine (FET) (165).

Targeting metabolic pathways has potential for conditioning the TME to become more responsive to frontline immunotherapies that have succeeded in more immunogenic cancers, as well as providing the opportunity to expand the limited treatment modalities that are currently approved. As experimental tools mature, our ability to better appreciate the heterogeneity between and within tumors advances. Many of the pathways mentioned have attracted study in more immunogenic cancers while preclinical data is sparse for glioblastoma models.

While there are a number of ongoing clinical trials exploring glioblastoma immunotherapies from the perspective of checkpoint blockade, there are relatively fewer trials pursuing immunometabolism modulation. For example, despite the extensive characterization of pathways like adenosine metabolism, glioblastoma research has yet to pursue A2aR inhibitors in the clinic. While the tryptophan metabolism pathway is by far the most clinically explored in IDH1 wildtype glioblastoma with 2 studies completed (NCT02052648, NCT02502708) and two studies recruiting (NCT04047706, NCT04049669), other pathways have unfortunately been less pursued. With the exception of one study pursuing arginine metabolism (NCT04587830) many of the other pathways analyzed in this review have not yet been explored in the clinic.

Metabolic mechanisms in glioma, and their interactions with the TME, and immune cells are helpful to develop precision medicine approaches. The presence of infiltrating immune cells in the TME presents a challenge but also a potential for therapeutic targets. Effector CD8+ T-cells express high levels of co-stimulatory and co-inhibitory molecules with a preferential accumulation of regulatory T cells (Tregs) in CNS tumors (166). The immunosuppressive environment of brain tumors has been highlighted in gliomas and other CNS tumors (144). Tregs play an essential role in ameliorating auto-immunity, but in the setting of brain TME, their anti-inflammatory activity creates a more permissive environment for tumor progression (167).

While targeting the IDH metabolic pathway with IDH inhibitors, and also more recently the IDH antigen, has demonstrated encouraging preliminary results in IDH mutated gliomas (95), glioblastoma or IDH wild type gliomas lack a uniformly expressed tumor specific antigen and are highly heterogenous. Research focus on targeting the metabolism in IDH wildtype glioblastoma, investigation of the role of metabolic pathways in glioblastoma, developing an appreciation for their differing activities across tumor types, and an increased willingness to explore these pathways in glioblastoma without first waiting for exploration in other tumors, should allow for selective and targeted treatment options and should inspire hope to treat patients with glioblastoma with immunotherapy.

## Conclusions

The field of immunometabolism represents a unique opportunity with emerging data supporting further research to fully understand mechanisms of resistance and to find potential synergy between immunometabolic pathways as well as other immunotherapy modalities. In addition to the pathways outlined, there remain other unknown metabolic aspects to discover to improve available therapies for patients with glioblastoma.

## Author Contributions

MK, AM, and JR conceived of and designed the work. AM, MK, WT, and AH-M drafted, and subsequently all authors revised the manuscript. KH and KS developed the figures, which were revised with input from all authors. All authors contributed to the article and approved the submitted version.

## Conflict of Interest

PF reports consulting for Monteris Medical. JS has an equity interest in Istari Oncology, which has licensed intellectual property from Duke related to the use of poliovirus and D2C7 in the treatment of glioblastoma. JS is an inventor on patents related to PEP-CMV DC vaccine with tetanus, as well as poliovirus vaccine and D2C7 in the treatment of glioblastoma. JS has an equity interest in Annias Immunotherapeutics, which has licensed intellectual property from Duke related to the use of the pepCMV vaccine in the treatment of glioblastoma. MK reports advisory roles for Janssen, AbbVie, and Jackson Laboratory for Genomic Medicine, and research funding from AbbVie, Bristol-Myers Squibb, and Specialized Therapeutics.

The remaining authors declare that the research was conducted in the absence of any commercial or financial relationships that could be construed as a potential conflict of interest.
